# A systematic review of gemcitabine and taxanes combination therapy randomized trials for metastatic breast cancer

**DOI:** 10.1186/2193-1801-3-293

**Published:** 2014-06-11

**Authors:** Qian Hu, Jun-xia Jiang, Long Luo, Xing Yang, Xiao Lin, Xiao-xiao Dinglin, Wei Zhang, Jun-yan Wu, He-rui Yao

**Affiliations:** Department of Oncology, Sun Yat-Sen Memorial Hospital of Sun Yat-Sen University, No. 107 West Yangjiang Road, Guangzhou, 510120 PR China; Department of Oncology, Yiyang Central Hospital of Hunan Province, No.232 West Wuyi Road, Yiyang, 413000 Hunan Province PR China; Department of Breast Tumor Center, Sun Yat-sen Memorial Hospital, Sun Yat-sen University, No. 107 West Yangjiang Road, Guangzhou, 510120 PR China; Pharmaceutical department, Sun Yat-sen Memorial Hospital, Sun Yat-sen University, No. 107 West Yangjiang Road, Guangzhou, 510120 PR China

**Keywords:** Metastatic breast cancer, Gemcitabine, Taxanes, Systematic review

## Abstract

**Purpose:**

Gemcitabine/taxanes-based combination shows anti-tumor activity for the treatment of metastatic breast cancer, but there is a debate regarding the advantages of gemcitabine and taxanes regimens as a first-line or second-line treatment for metastatic breast cancer. Here we conducted a systematic review and meta-analysis to compare the efficacy and toxicity for patients receiving chemotherapy with or without GT-based regimens.

**Methods:**

The randomized controlled trials were performed by searching Pubmed, MEDLINE, EMBASE, and conference proceedings. We identified eight randomized controlled trials and then extracted and combined the data using to calculate hazard ratios (HR). The primary outcomes were progression-free survival (PFS) and time to progression (TTP). The secondary outcomes were overall survival (OS) and acute toxicity. A meta-analysis was performed using Review Manager Version 4.2.

**Results:**

Eight eligible trails were identified. These studies involved 2234 patients with metastatic breast cancer, (1122 patients received GT-based combination regimen and 1112 patients received a regimen without the combination). A fixed-effects model meta-analysis showed that ORR and TTP are superior for GT-treated patients ORR (OR = 1.28, 95% CI 1.07-1.53), TTP (HR = 0.80; 95% CI 0.71-0.89). And GT-based combination significantly improved OS in the first-line subgroup (HR = 0.84; 95% CI 0.71-0.99). However, there were significant differences regarding acute hematological toxicity, particularly thrombocytopenia.

**Conclusion:**

Gemcitabine/taxanes-treated patients with metastatic breast cancer showed a significant improvement in the ORR, TTP and OS (first-line background) compared to patients not treated with the combination regimen.

## Introduction

Breast cancer is a major primary malignancy affecting the health of women, and the morbidity and mortality continue to increase in developed countries (Hortobagyi et al. 2005; Albain et al. 2005). Although overall survival (OS) has improved because of new therapeutic strategies in recent years, local recurrence or metastatic breast cancer (MBC) remains an incurable disease. The 5-year relative survival rate for MBC is only 27% (Jemal et al. 2008). The goals of MBC treatment are prolongation of survival while maintaining good quality of life and minimizing toxicity. Oncologists are searching for an optimal regimen with the best efficacy and safety to treat MBC patients. The first-line chemotherapy regimens for MBC are anthracycline based. However, as anthracycline containing regimens become the standard program in early breast cancer, most MBC patients have received anthracycline-based chemotherapy. Cardiotoxicity restricts the use of anthracyclines in MBC patients. New cytotoxic drugs have been substituted for anthracyclines, which reduce cardiotoxicity. The new drugs include the taxanes, such as paclitaxel and docetaxel; gemcitabine; capecitabine; and vinorelbine, which are administered either sequentially or in combination. Gemcitabine (Gemzar) is a nucleoside analog cytotoxic drug. Gemcitabine shows antitumor activity for a range of cancers both singly and in combination as a chemotherapy drug in phase II clinical trials (Blackstein et al. 2002; Yardley 2004; Markman et al. 2003). Several phase II clinical trials demonstrated that the overall response rate of single agent gemcitabine ranged from 15% to 38% (Blackstein et al. 2002; Hensley et al. 2002; Seidman 2001). There is an evidence that combination regimens are superior to sequential single regimens (Miles et al. 2002). Taxanes is good combination agent with gemcitabine, including paclitaxel and docetaxel.To define the combination regimen including gemcitabine in MBC, physicians have conducted phase III clinical trials to compare the efficacy and toxicity between regimens MBC (Albain et al. 2008; Chan et al. 2009; Joensuu et al. 2010; Zielinski et al. 2005; Levy & Fumoleau 2005; Brufsky et al. 2011; Nielsen et al. 2011; Papadimitriou et al. 2009).The combination of gemcitabine/taxanes-based(GT-based) combination is an effective regimen that is well tolerated with good response rates (Gudena et al. 2008). Previous studies have demonstrated that GT-based regimen has a clinically meaningful benefit (Joensuu et al. 2010; Nielsen et al. 2011; Papadimitriou et al. 2009). However, other data showed that the gemcitabine addition of taxanes was not associated with a statistically significant improvement in OR and TTP but did lead to increased toxicity (Joensuu et al. 2010; Nielsen et al. 2011; Papadimitriou et al. 2009). Thus, there is a debate regarding the advantages of GT-based regimens as a first-line or second-line treatment for MBC. We have conducted a systematic review and meta-analysis to estimate the benefits and risks of GT combination administration for MBC.

## Materials and methods

### Search methods

All published trials were eligible for inclusion in this study. We searched electronic databases (Pubmed, MEDLINE, EMBASE) in English by entering the following terms in the searching algorithm: (gemcitabine OR gemcitabine [Mesh] AND taxanes OR taxoid [Mesh] OR paclitaxel OR docetaxel) AND (breast tumors OR cancer of breast OR breast neoplasms [Mesh]) AND (advanced OR metastatic) AND (randomized controlled trial OR controlled clinical trial) OR randomized OR randomly OR trial). Additionally, we searched the Cochrane Central Register of Controlled Trials for randomized trials that compared gemcitabine treatment for MBC patients. The latest search was performed on September 31, 2013. We manually searched several oncology journals that publish clinical trials. The reference list of all articles was further searched for additional publications to broaden the search scope. The relevant articles and abstracts were selected and reviewed by two reviewers independently. We submitted the details of our systematic review “Gemcitabine-based chemotherapy for MBC: a systematic review and meta-analysis of randomized controlled trials” to the PROSPERO register. The registration number is CRD 42012002752.

### Eligibility criteria

The eligible clinical trials for inclusion were all controlled randomized studies that evaluated the efficacy and safety of GT-based chemotherapy for MBC patients. The studies included gemcitabine additional roles to taxanes and gemcitabine replacement roles to other non-taxane drugs. We selected double-blinded and randomized trials in addition to non-blinded studies because of the difficulty of blinding for GT administration. The articles selected were completed trials published in full papers or abstracts. All unpublished and ongoing reports were excluded. Trials with two arms were included and one-arm articles and three-arm were excluded. We accepted trials with first-line and second-line metastatic or advanced breast cancer patients. The studies of early stage breast cancer were excluded. All cytotoxic chemotherapy regimens were considered eligible for the meta-analysis, and new targeted drugs such as bevacizumab were included. The trials that compared single agent and other cytotoxic drugs were excluded. The studies based on phase I trials and single-arm data phase II trials were excluded. The reported outcomes included time to progression (TTP), progression-free survival (PFS), overall survival (OS) and the drug toxicity. However, economic evaluations and quality-of-life (QoL) were not considered. Single gemcitabine trials were excluded from our study. Additionally, data of interim analyses were excluded only when we retrieved the complete results from the same study. When we were uncertain of the eligibility of a trial, we discussed its eligibility until a decision was reached.

### Data extraction

We recorded the following data from each report: first author’s name, publication journal name, year of publication, and number of patients. We also extracted the following individual patient characteristics: age, performance status, ER status, PR and HER-2 status and prior therapy. The following information for each randomized trial was requested: allocated treatment, number of withdraws per arm, blinding, regimen details, clinical outcomes, and study quality. All the information was extracted from each study by Hu and Jiang independently using the same recording forms. The data were then reviewed by Yao. When there was a discrepancy, we reached a consensus by discussion. The primary ends included the total response (CR + PR) using the 4-point WHO scale, time to progression (TTP), progression-free survival (PFS) and overall survival (OS). The secondary end points were acute toxicity including grade 3/4 anemia, neutropenia, febrile neutropenia, fatigue, liver function impairment, nausea/vomiting, and neuropathy graded using WHO criteria. The HRs reported with 95% CIs were extracted when possible. We could not obtain the HR and 95% CI from one of the studies. In such cases, we estimated the P-value of the log-rank statistics by examining the survival curves. The survival curves were enlarged to minimize the reading bias. All data were examined for missing values, and patient follow up was assessed to ensure it was well balanced. If we could not combine the data from the articles because of low numbers of trials and incomplete trials, we performed a systematic analysis of the data.

### Quality assessment

We used the Jadad scale to assess trial quality. The following items details were extracted: blinding; method of randomization-including stratification factor-number of patients participants randomly assigned; excluded from analysis by arm; patientparticipants’ follow-up time (if possible) by arm; number of patients participants lost to follow-up by arm.

### Statistical analysis

All the randomized assigned patients were included in the analyses according to the allocated treatment. The data extracted from the trials were entered into the Cochrane Collaboration software (RevMan version 4.2; http://www.cochrane.org). Using the fix random-effects model of DerSimonian and Laird (DerSimonian & Laird 1986), the log hazard ratios (HR) and their variances for time-to-event data were estimated using published methods. Appropriate summary statistics or Kaplan-Meier curves were reported when possible. The results of each trial including HRs and 95% CIs were combined using standard meta-analytic methods to estimate an overall effect for the MBC patients treated with or without GT-based combination. The HRs reported with 95% CIs were extracted when possible. We could not obtain the HR and 95% CI from one of the studies so we estimated by the P-value of the log-rank statistics by examining the survival curves. The survival curves were enlarged to minimize the reading error. We used X^2^ statistics to assess the between-study heterogeneity. We also calculated the I^2^ statistic expressing the proportion of variability in the results. To assess publication bias, we used a funnel plot.

We submitted the details of our systematic review “Gemcitabine-based chemotherapy for MBC: a systematic review and meta-analysis of randomized controlled trials” to the PROSPERO register. The registration number is CRD 42012002752.

## Results

**Figure 1:**
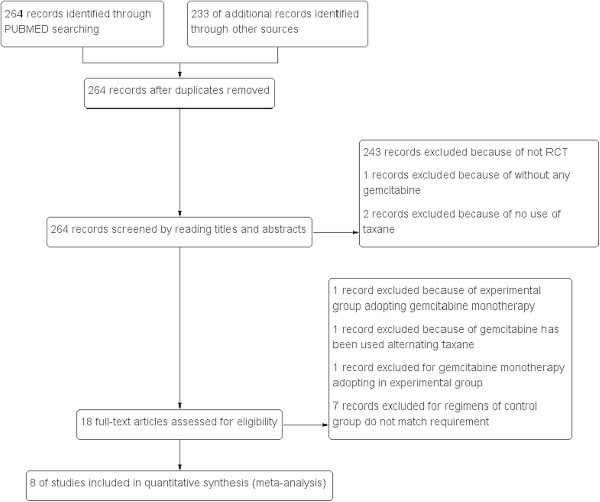
**Flow chart of study selection.**

The flow chart of our study is shown in Figure 1. Both reviewers agreed to include eight trials involving a total of 2234 female patients with MBC in the meta-analysis.

### Characteristics of the selected trials

The results of literature search in our study are shown in Table 1. Eighteen studies were identified by evaluating the title and abstract. After reading the full text, one article was excluded because gemcitabine was administered separately. One article was excluded because gemcitabine has been used alternating taxanes. One article was excluded because gemcitabine monetherapy adopting in experimental group. Senven articles were excluded because the control group regimens were not match requirement. Finally, eight studies including 2234 patients were identified. According to prior selection criteria, eight prospective and randomized trials were selected for inclusion in this study. The clinical characteristics were matched for performance status, age, and tumor stage. All studies reviewed were considered high quality. The patients eligible for these studies had proven histological or cytological MBC with the same baseline data and without evidence of selection bias. All of the eight trials are well organized, rigorous, and prospective randomized controlled trials. The OS, PFS, TTP, ORR and toxicity data of patients were extracted from eight trials.

**Table 1:** **Characteristics of included studies**

Study ID	Arms	Patients	Treatments (cycle)	Endpoints	Study design	Lose	Treatment lines	Jada scale
**Dorte L. Nielsen** 2011	Gemcitabine + Docetaxel	170	G 1,000 mg/m2 d1,8 + D 75 mg/m2 d8(21d)	OS.ORR.TTP.toxicity	phase3, random,open-label	6	First or second-line	3
	Docetaxel	167	D 100 mg/m2 d1(21d)					
**Kathy S. Albain** 2008	Gemcitabin + Paclitaxel	266	G 1,250 mg/m2 d1,8 + P 175 mg/m2 d1(21d)	OS.TTP.ORR.toxicity	phase3, random,unclear	8	first-line	3
	Paclitaxel	263	P 175 mg/m2 d1(21d)					
**H. Joensuu** 2010	Docetaxel + Gemcitabin(alternating)	122	D l100 mg/m2 d1 + G 1000 mg/m2 d1,8(21d)	TTP.OS.ORR.toxicity	phase3, random,open-label	3	first-line	3
	Docetaxel	115	D l100 mg/m2 d1(21d)					
**Christos A. Papadimitriou** 2009	Gemcitabin + Docetaxel	41	D 35 mg/m2 + G 600 mg/m2(7d)	ORR.OS.TTP.toxicity	phase2, random, unclear	13	second-line	2
	Docetaxel	34	D 40 mg/m2(7d)					
**Adam Brufsky** 2011	Gemcitabin + Paclitaxel + Bevacizumab	93	P 90 mg/m2 d1, 8, 15 + B 10 mg/kg d1,15 + G 1500 mg/m2 d1, 15(28d)	ORR.PFS.OS.toxicity	phase2, random, open-label	28	first-line	3
	Paclitaxel + Bevacizumab	94	P 90 mg/m2 d1, 8, 15 + B 10 mg/kg d1, 15(28d)					
	Vinorelbine	127	V 30 mg/m2 d1,8(21d)					
**C. Levy** 2005	Gemcitabin + Docetaxel	153	D 75 mg/m2 d1 + G 1000 mg/m2 d1, 8(21d)	ORR.PFS.TTP.toxicity	Phase3,random,unclear	Unknown	second-line	2
	Capecitabine + Docetaxel	152	D 75 mg/m2 d1 + C 1250 mg/m2 bid d1-14(21d)					
**Zielinski** 2005	Gemcitabine + epirubicin + and paclitaxel(GET)	124	G 1,000 mg/m2 d1, 4 + E 90 mg/m2 d1+ P 175 mg/m2	TTP.ORR.toxicity	Phase3,random,unclear	Unknown	first-line	3
			d1(21d)					
	Fluorouracil + Epirubicin + Cyclophosphamide(FEC)	135	F 500 mg/m2 d1 + E 90 mg/m2 d1 + C 500 mg/m2 d1(21d)					
**Stephen Chan** 2009	Gemcitabin + Docetaxel	153	G 1000 mg/m2 d1,8 + D 75 mg/m2 d1(21d)	PFS.ORR.OS.toxicity	Phase3,random,unclear	8 + 3	first + second-line	3
	Capecitabine + Docetaxel	152	C 1,250 mg/m2 bid d1-14 + D 75 mg/m2 d1(21d)					

G = gemcitabine, D = docetaxel, C = capecitabine, F = flurouracil, C = cyclophospham, E = epirubicin, P = paclitaxel, V = vinorelbine, B = bevacizumab, OS = overall survival, ORR = objective response rates, PFS = progression-free survival, TTP = time to progression.

### Objective response rates

**Figure 2:**
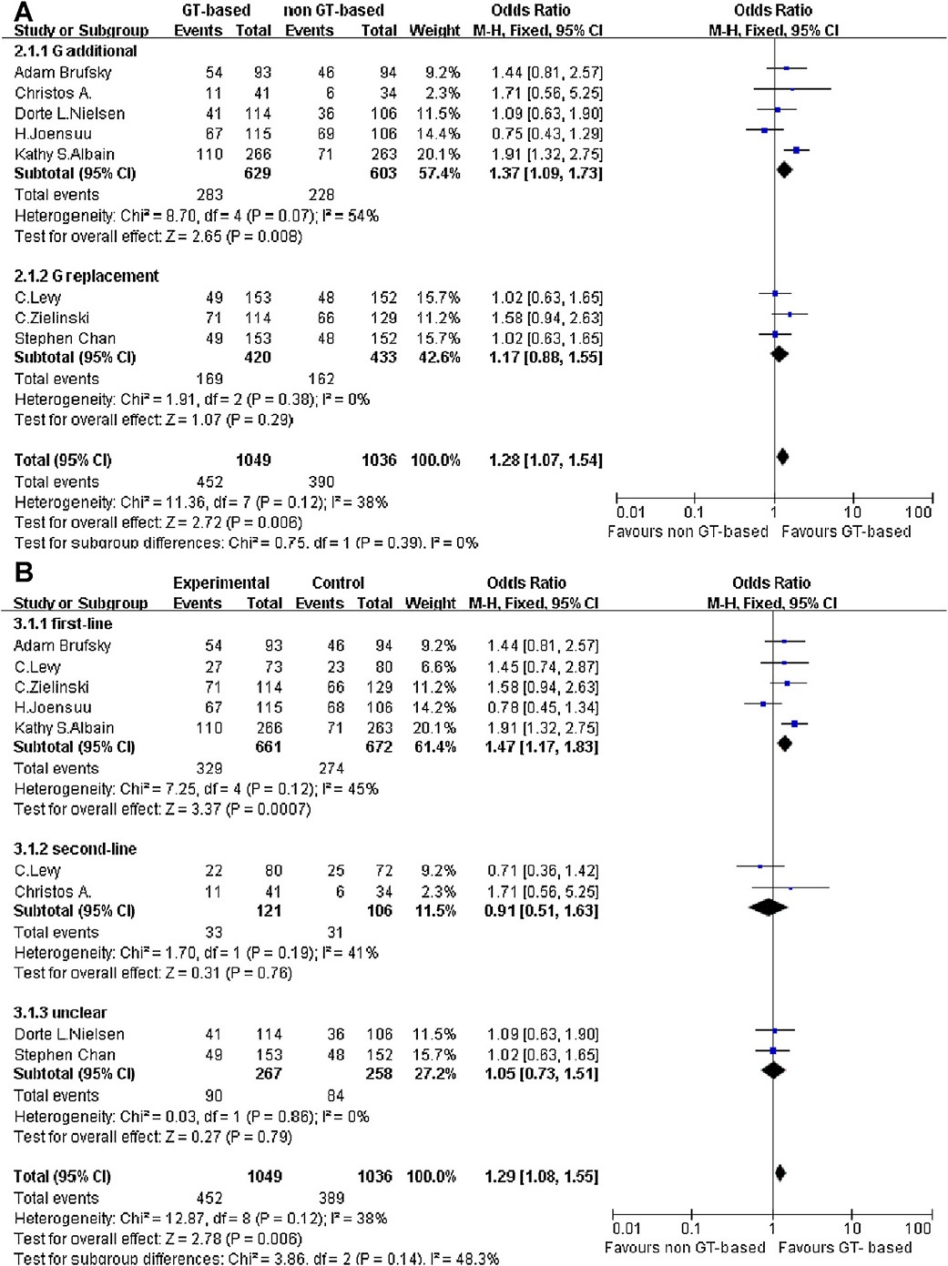
**Response of GT-based combination therapy versus non GT-based combination for metastatic breast cancer.** ORR were analyzed with the fixed effect model. (**A**: Meta-analysis of ORR, subgroup: gemcitabine role; **B**: Meta-analysis of ORR, subgroup: therapy lines).

The ORR were reported for all trials and accounted for 2234 events. Subgroups were established for the therapy lines and the gemcitabine roles. Based on the total data, there was no evidence of heterogeneity (P = 0.16,I^2^ = 34%) among trials as shown in Figure 2. The results demonstrated GT-based therapy increases ORR, (OR = 1.28, 1.07 to 1.53, P = 0.006). It showed that GT-based regimen got benefit in first-line background as ORR (OR = 1.47, 1.17 to 1.83, P = 0.0007). However, there was no significant difference for ORR (OR = 0.91, 0.51 to 1.63, P = 0.76 ) in second-line subgroup. And the analysis results showed there was benefit for GT-based chemotherapy on ORR (OR = 1.37, 1.09 to 1.73, P = 0.008; 1.17, 0.88 to 1.55, P = 0.29) in gemcitabine additional roles and gemcitabine replacement subgroups.

### Progression-Free Survival (PFS)

**Figure 3:**
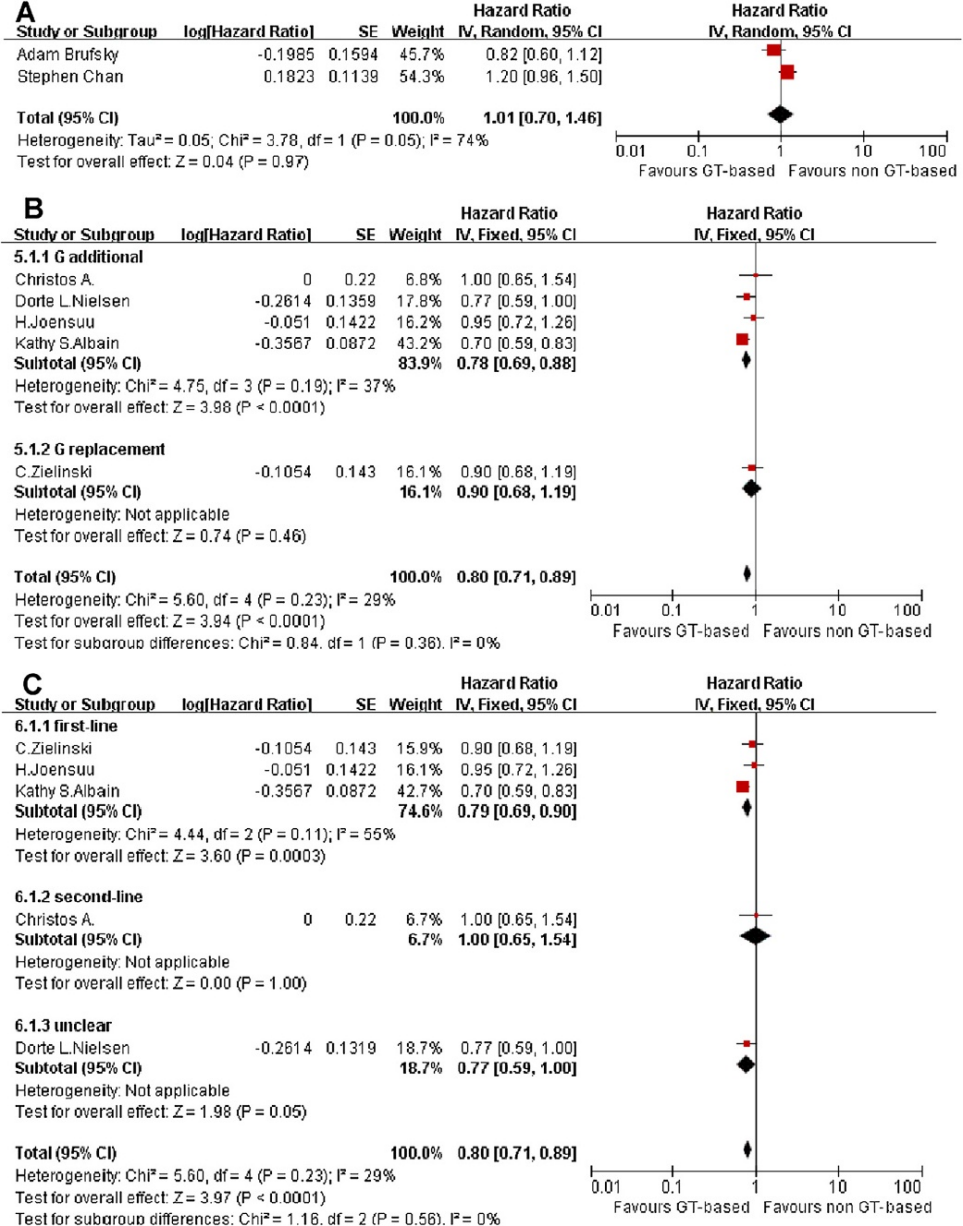
**Effect of GT-based combination therapy versus non GT-based combination for MBC.** (**A**: Meta-analysis of PFS; **B**: Meta-analysis of TTP, subgroup:gemcitabine role; **C**: Meta-analysis of TTP, subgroup:therapy lines).

Only two studies were identified as shown in Figure 3. There was significant heterogeneity found in the data (P = 0.05, I^2^ = 74%). Heterogeneity may be caused by a few trial numbers or small samples, which were not eliminated. Therefore, a random effect model was selected. The meta-analysis result shows that PFS was not significantly improved (HR = 1.01, 0.7 to 1.46, P = 0.47).

### Time to Progression (TTP)

TTP was reported in five articles as shown in Figure 3. There was no evidence of heterogeneity (P = 0.23, I^2^ = 29%) among trials. HR for single studies ranged from 0.70 to 1.00. GT-based treatment obviously prolong TTP (HR = 0.80, 0.71 to 0.89, P < 0.0001). However, in the subgroup study the gemcitabine additional role group shows the significant difference (HR = 0.78, 0.69 to 0.88, P < 0.0001). Gemcitabine replacement chemotherapy subgroup contains only one study shows no significant difference (HR = 0.90, 0.68 to 1.19). Also, the first-line subgroup study shows GT- based treatment obviously prolong TTP (HR = 0.79, 0.69 to 0.92, P = 0.0003).

### Overall Survival (OS)

**Figure 4:**
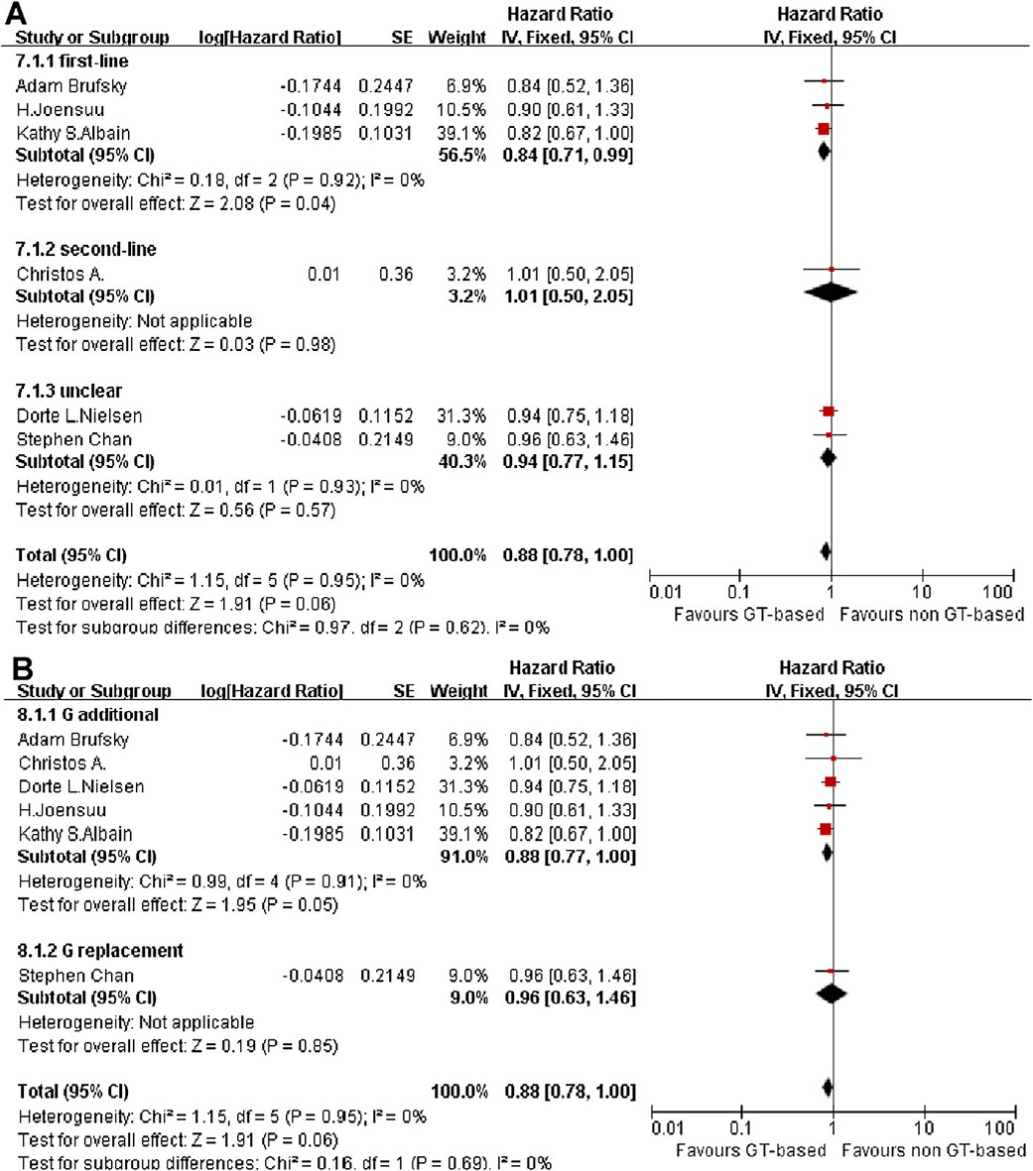
**Effect of GT-based combination therapy versus non GT-based combination for MBC.** HR were analyzed with the fixed effect model. (**A**: Meta-analysis of OS, subgroup: therapy lines; **B**: Meta-analysis of OS, subgroup: gemcitabine role).

OS data were taken from seven articles which were defined as first-line, second-line and unclear subgroups as shown in Figure 4. There was no statistically significant heterogeneity in the hazard ratios (HRs) for overall survival from the individual trials (P = 0.95, I^2^ = 0%). GT-based chemotherapy had no significant difference compared to other regimens (HR = 0.88, 0.78 to 1.00, P = 0.06). However, patients benefited from GT-based combination therapy as a first-line chemotherapy (HR = 0.84, 0.71 to 0.99, P =0.04).

### Toxicity

**Figure 5:**
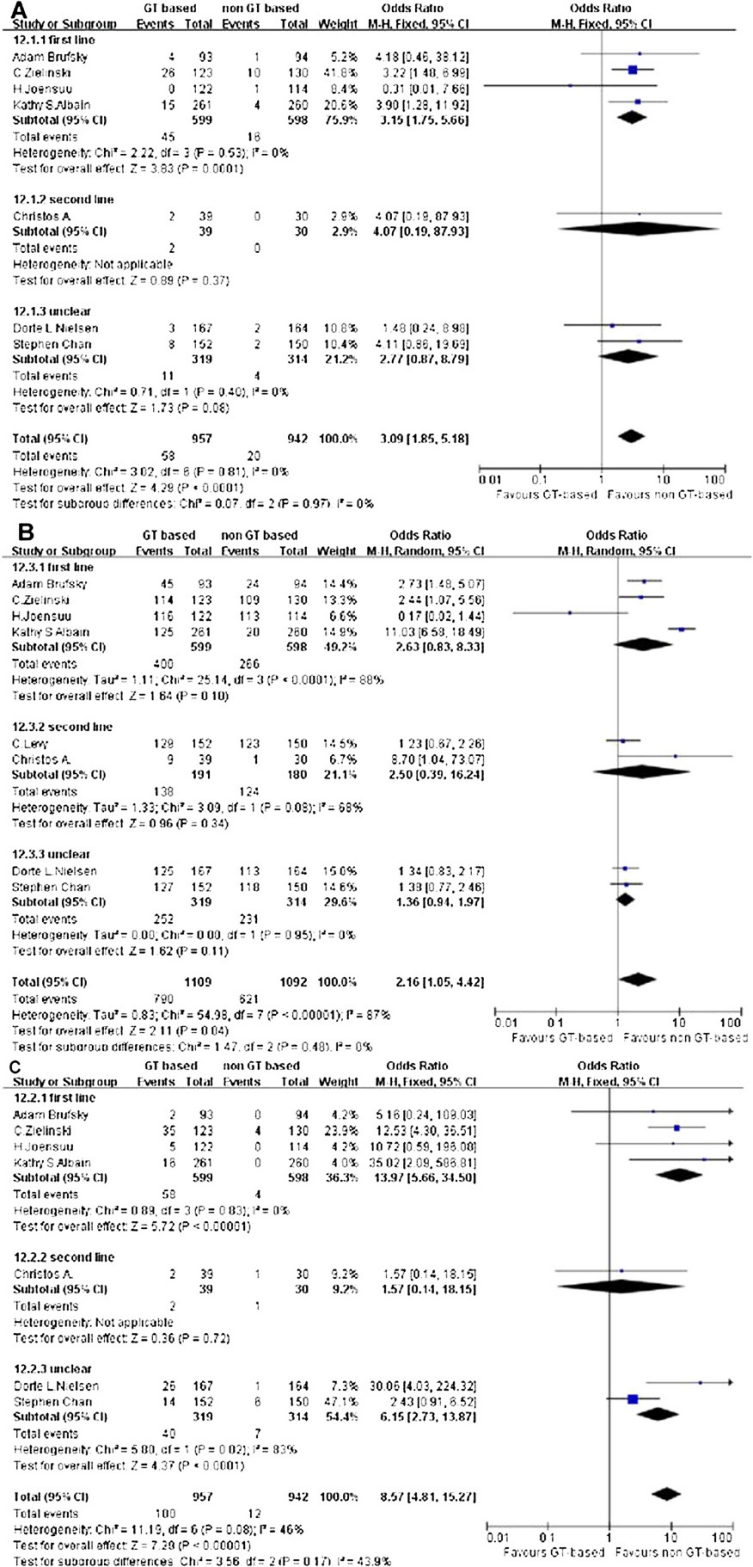
**Side effect of GT-based combination therapy versus non GT-based combination for MBC.** (**A**: anemia; **B**: neutropenia; **C**: thrombocytopenia subgroup: therapy lines).

Reports of hematologic toxicity were variable across the trials as shown in Figure 5. All toxicity mentioned below were only calculated grade 3–4. The data on anemia and thrombocytopenia were reported in seven studies. Neutropenia was reported for all trials. Most of these data were homogeneous except for the neutropenia analysis The number of patients experiencing grade 3–4 hematologic toxicity was greater in the GT-based arm. The odds ratio for single studies with anemia analysis ranged from 0.31 to 4.18. There was only one study with an OR < 1, (OR = 3.09, 1.89 to 5.18, P < 0.0001). The odds ratio for single studies with neutropenia analysis ranged from 0.34 to 11.03, and only one study had OR < 1, (OR = 2.17, 1.07 to 4.38, P = 0.03). The odds ratio for single studies on thrombocytopenia ranged from 1.57 to 35.02. There were no studies with OR < 1, (OR = 8.57, 4.81 to 15.27, P < 0.00001). Based on those data, thrombocytopenia is a problem in GT-based chemotherapy. And in the first-line subgroup.

### Sensitivity analysis

Due to the high heterogeneity in the above analysis, we performed subgroup analysis in the meta-analysis. A sensitivity analysis was also conducted by removing one study at a time and calculating the pooled HRs for the remaining studies. We found that no article substantially influenced the pooled result in this analysis.

### Publication bias

The publication bias was evaluated by a funnel plot. The funnel plot shapes showed no obvious evidence of asymmetry. The results suggested that publication bias was not evident in this meta-analysis.

## Discussion

Although the therapy for MBC has improved dramatically over the past decade, cancer mortality among women continues to be major concern. When the disease is incurable, prolonging the life span and relieving pain are still goals for oncologists. Gemcitabine has been shown to be active as a single agent in MBC with good tolerability (Tripathy 2002; Spielmann et al. 2001). Gemcitabine rarely induces severe gastrointestinal events or symptomatic cardiac events but does cause hematologic toxicity (Green 1996). Taxanes are currently introduced early in MBC treatment, in patients with no or minimal prior anthracycline exposure and/or in combination with anthracyclines and gemcitabine (von Minckwitz et al. 2013). Several studies have investigated the impact of using drug combinations involving GT for the treatment of MBC. There were eight randomized controlled trails eligible in our study. While searching for studies we found case report and non-randomized trial with GT-based combinations for MBC (Kakimoto et al. 2012; Delfino et al. 2004).

The meta-analysis showed that GT-based combination chemotherapy regimens resulted in a significant benefit for ORR, TTP and OS (first-line background). Gemcitabine is an effective agent that is well tolerated with good response rates, we found the same results in non-small cell lung cancer and pancreatic cancer (Heinemann et al. 2008; Russo et al. 2009). These results indicate the major benefit from gemcitabine occurs in additional and replacement chemotherapy for cancer patients. Heinemann revealed a significant survival benefit in advanced pancreatic cancer for gemcitabine plus cytotoxic agents compared to single agent gemcitabine in advanced pancreatic cancer (Heinemann et al. 2008). Russo found a significant difference in ORR favoring gemcitabine-based doublets over single agents (Russo et al. 2009). Gemcitabine-based regimens appeared to be effective and feasible compared with other cytotoxic agents in the treatment of the cancer. Recent data have focused on the role of gemcitabine in MBC. The recent meta-analysis indicated that gemcitabine-based chemotherapy was as effective as gemcitabine-free chemotherapy in patients with MBC with increased hematological toxicity (Li et al. 2013). However, combination regimens are superior to sequential single regimens (Seidman 2001). Furthermore, we have to optimize the combination agent with gemcitabine.

Our findings clearly support the use of MBC treatment with GT-based chemotherapy and provide better data than previous single trials and three review articles. The single trials produce unclear results that include both false-negative or false-positive data. The role of GT-based combinations in MBC treatment remains controversial (Gudena et al. 2008; Colomer 2004). The third review indicate GT combinations represented a viable alternative to currentlyaccepted taxane combinations such as capecitabine/docetaxel (Dent et al. 2008). However, these studies were qualitative and not quantitative. Additional data are needed to identify the role of GT-based combination in the treatment of MBC. A quantitative meta-analysis may allow evaluation of the average effect of gemcitabine-based combination treatment. A meta-analysis can also assist in identifying causes of heterogeneity. There is no meta-analysis published regarding the efficacy and safety of gemcitabine/taxenes-based combinations in MBC patients. Our findings suggest that gemcitabine-based chemotherapy may provide better efficacy.

Additional studies similar to our review are required to better estimate the short-term and long-term efficacy conferred by the optimal chemotherapy regimen for the MBC management. Another strength of our study is a comprehensive search with two reviewers reading each article independently. The relevant articles and abstracts were selected and reviewed by Hu and Jiang. When we were uncertain about the eligibility of a trial, we would discuss the trial to reach a conclusion. Furthermore, our review includes high quality trials with GT-based-based combination regimens of MBC analyzed by meta-analysis.

There are limitations of our study that deserve comment. The heterogeneity of GT-based chemotherapy regimens in the studies may have led to an underestimation of our results and led to a hazard estimate closer to the null. To avoid these defects we established subgroups in the meta-analysis to discriminate the heterogeneity. The eligible randomized controlled trials were based on different settings including first-line and second-line treatment. After carefully identifying each regimen in all eight articles, we found that there were two types of comparisons in the eligible RCTs. There were regimens that used gemcitabine as an add-on treatment and regimens that used gemcitabine as a replacement drug. Thus, we divided the studies into the additional gemcitabine group and a gemcitabine replacement group, also first-line, second-line and unclear group. In addition, we extracted the relative data not the absolute data such as the OS in the eligible studies to minimize the heterogeneity for the different settings. Second, there was heterogeneity in the length of follow up in the long-term mortality studies. Third, some of our selected studies are not blinded. As a result, high-quality randomized, multicenter, blinded, controlled trials are still required.

One major limitation is the number of trials is quite small and may not represent the real situation. However, the number of patients is 2234.

In conclusion, we demonstrated that GT-based combination treated patients showed a significant improvement in the ORR and TTP in patients with MBC compared with non GT-based treated patients. GT-based regimens led to more serious hematologic toxicity. The therapy line subgroup analysis demonstrated that GT-based combination chemotherapy improved ORR, TTP and OS in the first-line background; while in the second-line background GT-based combination chemotherapy showed benefit of ORR,but did not show any benefit in TTP and OS. And the gemcitabine role subgroup analysis demonstrated that GT-based combination therapy may well be superior to non GT-based in gemcitabine additional therapy group. Therefore, the results from our meta-analysis imply that GT-based chemotherapy showed benefit especially for first-line background. Additional prospective clinical trials are warranted to evaluate treatment combinations using GT-based.
